# Multi-parameter MRI radiomics model in predicting postoperative progressive cerebral edema and hemorrhage after resection of meningioma

**DOI:** 10.1186/s40644-024-00796-3

**Published:** 2024-11-01

**Authors:** Kangjian Hu, Guirong Tan, Xueqing Liao, Weiyin Vivian Liu, Wenjing Han, Lingjing Hu, Haihui Jiang, Lijuan Yang, Ming Guo, Yaohong Deng, Zhihua Meng, Xiang Liu

**Affiliations:** 1 Department of Radiology, The Affiliated Yuebei People’s Hospital of Shantou University Medical College, Shaoguan, Guangdong Province China; 2 Advanced Neuroimaging Laboratory, The Affiliated Yuebei People’s Hospital of Shantou University Medical College, Shaoguan, Guangdong Province China; 3 GE Healthcare, MR Research China, Beijing, China; 4 Yanjing Medical College, Capital Medical University, Beijing, China; 5 Department of Neurosurgery, Peking University Third Hospital, Peking University, Beijing, China; 6 Department of Neurosurgery, The Affiliated Yuebei People’s Hospital of Shantou University Medical College, Shaoguan, Guangdong Province China; 7 Department of Research & Development, Yizhun Medical AI Co. Ltd, Beijing, China; 8 Advanced Neuroimaging Laboratory, Department of Radiology, The Affiliated Yuebei People’s Hospital of Shantou University Medical College, Shaoguan, Guangdong Province China

**Keywords:** Radiomics, Meningioma, Machine learning, Hemorrhage, Cerebral edema

## Abstract

**Background:**

Postoperative progressive cerebral edema and hemorrhage (PPCEH) are major complications after meningioma resection, yet their preoperative predictive studies are limited. The aim is to develop and validate a multiparametric MRI machine learning model to predict PPCEH after meningioma resection.

**Methods:**

This retrospective study included 148 patients with meningioma. A stratified three-fold cross-validation was used to split the dataset into training and validation sets. Radiomics features from the tumor enhancement (TE) and peritumoral brain edema (PTBE) regions were extracted from T1WI, T2WI, and ADC maps. Support vector machine constructed different radiomics models, and logistic regression explored clinical risk factors. Prediction models, integrating clinical and radiomics features, were evaluated using the area under the curve (AUC), visualized in a nomogram.

**Results:**

The radiomics model based on TE and PTBE regions (training set mean AUC: 0.85 (95% CI: 0.78–0.93), validation set mean AUC: 0.77 (95%CI: 0.63–0.90)) outperformed the model with TE region solely (training set mean AUC: 0.83 (95% CI: 0.76–0.91), validation set mean AUC: 0.73 (95% CI: 0.58–0.87)). Furthermore, the combined model incorporating radiomics features, and clinical features of preoperative peritumoral edema and tumor boundary adhesion, had the best predictive performance, with AUC values of 0.87 (95% CI: 0.80–0.94) and 0.84 (95% CI: 0.72–0.95) for the training and validation set.

**Conclusions:**

We developed a novel model based on clinical characteristics and multiparametric radiomics features derived from TE and PTBE regions, which can accurately and non-invasively predict PPCEH after meningioma resection. Additionally, our findings suggest the crucial role of PTBE radiomics features in understanding the potential mechanisms of PPCEH.

**Supplementary Information:**

The online version contains supplementary material available at 10.1186/s40644-024-00796-3.

## Background

Meningioma is the most common intracranial tumor [1]. Its incidence in the United States from 2016 to 2020 was 9.73 per 100,000 population, accounting for 40.8% of central nervous system (CNS) tumors and 56.2% of non-malignant CNS tumors [2]. At present, surgical resection is the first-line treatment [3]. The postoperative complications include wound infection, hemorrhage, cerebral spinal fluid leak, cerebral edema, new seizure, and neurological deficits, et al. [4].

Among them, postoperative progressive cerebral edema and hemorrhage (PPCEH) are major surgical complications [5]. In the literature, the incidence of postoperative hemorrhage ranged from 6 to 33% [6, 7], and the incidence of postoperative cerebral edema was reported from 11 to 44% [8, 9]. The mechanism of PPCEH is complex, and some results are still in controversy [4, 9].

PPCEH can result in refractory intracranial hypertension, severe and persistent neurological deficits, prolonged hospitalization, and deterioration of quality of life in patients with meningioma [8]. The aggravation of PPCEH was associated with a higher mortality [10]. Given the prognostic and therapeutic importance, there is a compelling need to build a comprehensive model to predict the risk of PPCEH.

Radiomics, a powerful tool, which can extract high-throughput and quantitative imaging features from medical images to explore the relationship between biomedical tissue characteristics and clinical value information [11]. In the field of meningioma, radiomics has exhibited promising potential in tumor segmentation, preoperative tumor grading, differential diagnosis between meningioma and other brain tumors, prediction of tumor biological characteristics including histology subtypes, brain invasion, ki-67 index, and tumor consistency, prediction of treatment response after radiosurgery, prognostic implications of recurrence and overall survival [12]. Most of these radiomics studies focused on the MRI features derived from tumor enhancement (TE) regions, few studies investigated the features of peritumoral brain edema (PTBE) regions, which can be developed in up to 78% of patients with meningioma. In addition, preoperative PTBE has been demonstrated to have a significant association with the presence of preoperative cognitive deficits, incidences of seizure, and postoperative complications [13].

Therefore, we hypothesize that the combination of radiomics features from both TE regions and PTBE regions will provide better value in the prediction of PPCEH. In the current study, our purpose is to develop and validate a multiparametric MRI machine learning radiomics model in predicting PPCEH after total resection in patients with meningioma.

## Materials and methods

### Patient selection

This retrospective study was approved by the ethics committees of the Yuebei People’s Hospital, and the requirement for informed consent was waived. We reviewed 207 cases in our institution with pathologically confirmed meningioma on surgical resection, from January 2018 to March 2023. Patients were included in the study based on the following enrollment criteria: (1) preoperative brain MRI examination. (2) no radiotherapy, chemotherapy, or any other treatment before the MRI examination. (3) postoperative continuous follow-up CT and or MRI examinations within one month after surgical resection. (4) complete clinical data. Exclusion criteria were: (1) abnormal coagulation function. (2) Patients with other significant diseases affecting brain imaging results, such as severe cerebrovascular diseases. (3) unsatisfactory preoperative images, including severe artifacts, or incomplete MRI examination.

**Fig. 1:**
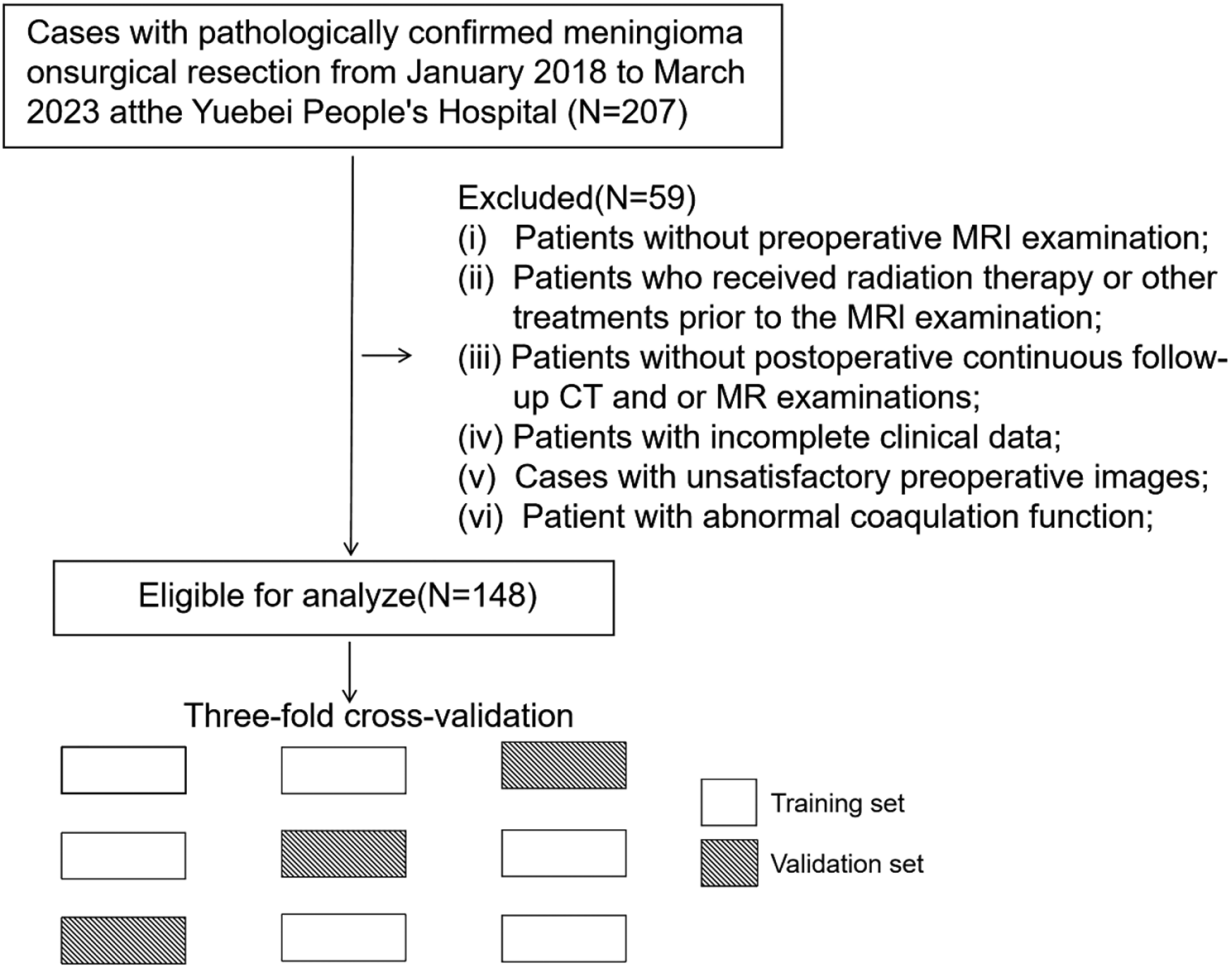
The flowchart for patient enrollment and the stratified three-fold cross-validation process

**Fig. 2:**
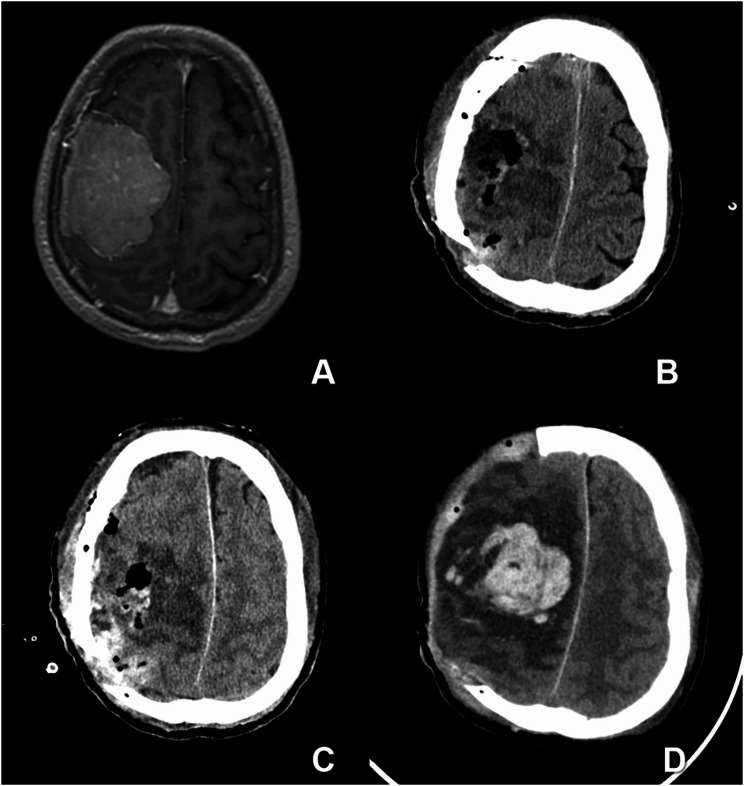
A 45-years old patient with postoperative progressive hemorrhage after right-frontal meningioma resection. (**A**) post-contrast T1WI before surgical rection, (**B**) CT examination immediately after surgical rection, (**C**) post-operation CT 6 h later, and (**D**) Follow-up CT two days later

**Fig. 3:**
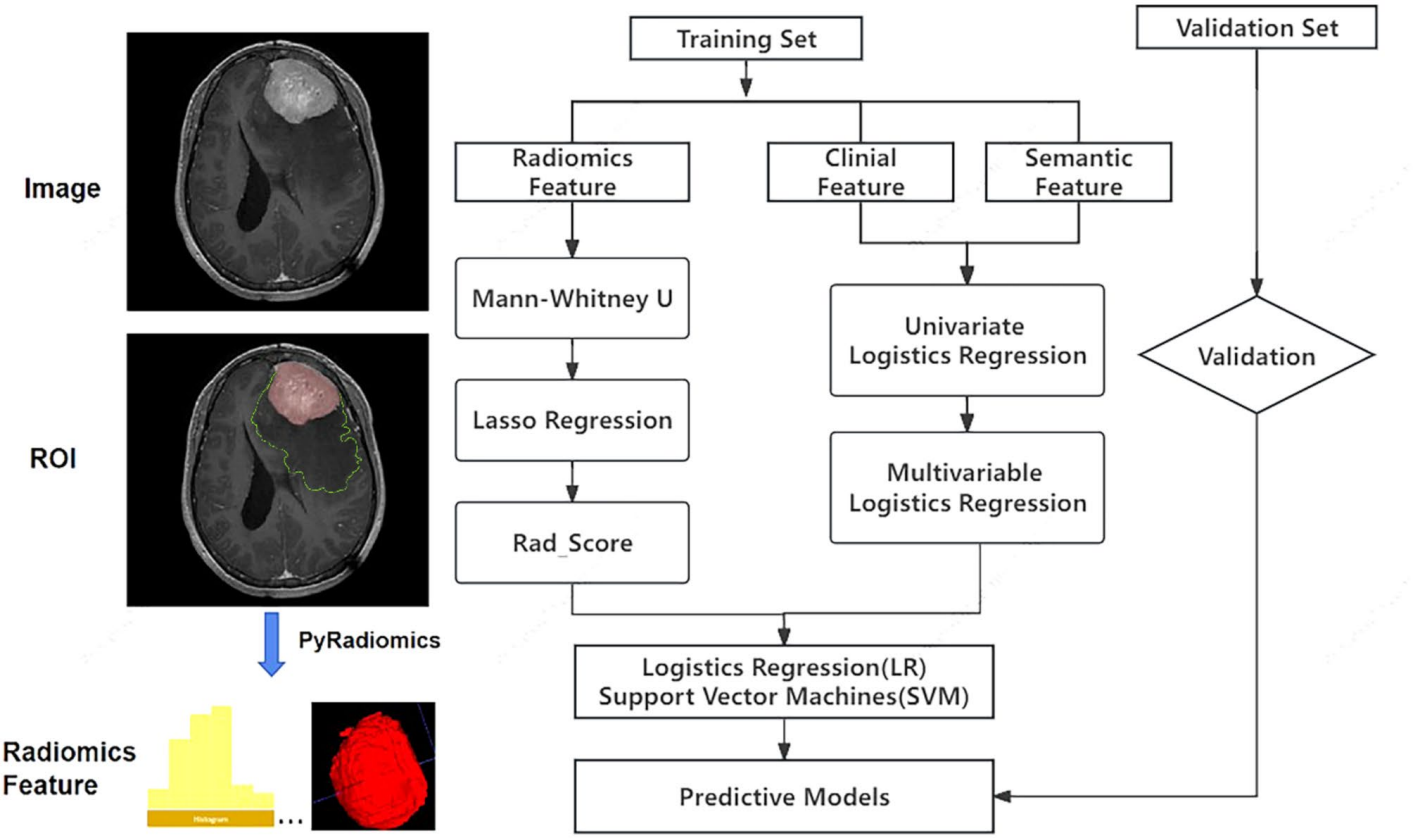
The overall workflow of radiomics processing and predictive-models construction

After patient selection, the dataset was split using a stratified three-fold cross-validation approach to ensure robust and unbiased model training and validation. In this method, the data were divided into three subsets, with each subset being used once as the validation set while the remaining two subsets were used for training. This iterative process was designed to enhance the model’s generalizability and performance assessment. A flow diagram of the patient inclusion process is shown in Fig. 1 (see Figs.  2,  3).

### Clinical and pathologic characteristics

Clinical data were obtained from medical and operative reports, including age, gender, hypertension (negative or positive), blood pressure classification (systolic blood pressure less than 140 mmHg and diastolic blood pressure less than 90 mmHg is considered normal. Systolic blood pressure greater than 140, 160, or 180 mmHg, and diastolic blood pressure greater than 90, 100, or 110 mmHg, correspond to stages 1, 2, and 3 hypertension, respectively), HbA1c, glucose, hyperlipidemia (negative or positive), epilepsy(negative or positive), tumor location (sellar region, supratentorial or infratentorial), intraoperative blood loss, tumor adhesion (no adhesion / mild adhesion, severe adhesion, invasion of bone / venous sinuses), and tumor shape (round / globular, lobulated or irregular), preoperative peritumoral edema (negative or positive), and tumor margin (well-defined margin / partially defined margin / poorly defined margin / infiltration into surrounding tissues).

The histopathology grades of meningioma samples were reviewed according to the criteria of the 2021 WHO Classification of Tumors of the Central Nervous System. The Ki-67 proliferation index was recorded.

#### The definition standard for PPCEH

1. Progressive cerebral edema can be defined if it meets any of the following criteria [9]: (1) New sheet or finger brain edema occurs after operation, and the maximum diameter of edema is not less than 2 cm; (2) If there is no PTBE before operation, flaky, finger shaped or annular brain edema occurs after operation, and the maximum diameter of the tumor cavity in the same layer of the tumor or operation area is not less than 2 cm before operation or on the first day after operation. (3) If there is PTBE before operation, the maximum diameter of lamellar, finger like, or annular brain edema after operation is not less than 2 cm compared with the maximum diameter of brain edema on the same plane before operation or on the first day after operation. (4) Postoperative repeated CT and or MRI examinations show increased cerebral edema.
2. Progressive cerebral hemorrhage can be defined if it meets any of the following criteria: (1) The amount of bleeding in the surgical area is greater than 50 ml. (2) Postoperative repeated CT and or MRI examinations show increased cerebral hemorrhage. (3) Patients who require a second surgery for hematoma removal or decompressive craniectomy treatment. (4) The clinical condition deteriorates, with dysfunction of consciousness, unequal pupil size, and even death.

### Image acquisition

Preoperative brain MRI was performed using a clinical scanning protocol on five MRI scanners (GE Signa HDxt, GE Discovery MR750w, GE Discovery MR750, Siemens Symphony, and Siemens MAGNETOM Vida). T1-weighted imaging (T1WI), contrast-enhanced T1-weighted imaging (CE-T1WI), diffusion-weighted imaging (DWI), apparent diffusion coefficient (ADC) maps were derived from DWI with b = 0 and 1000 s/mm^2^, and axial T2-weighted imaging (T2WI) were acquired and used in our study. Detailed parameters of MRI scanners, sequences, and the number of subjects scanned are listed in Supplementary Table 1. Postoperative cranial CT was performed with 64-slice and 128-slice GE scanners.

### Segmentation and feature extraction

The publicly available software, ITK-SNAP (version 3.8.0, http://www.itksnap.org), was selected for the segmentation tasks. A radiologist with 6 years of experience performed the segmentation of images of all patients. A senior researcher with 30 years of clinical expertise in neuroimaging examined all segmentations. During the segmentation and examination processes, researchers were blinded to the characteristic features of the subjects.

#### Region segmentation

The TE region was segmented on T1WI, CE-T1WI, T2WI, and ADC. The ROIs should be selected as close to the tumor edge as possible, from which areas with edema, necrosis, calcification, and peritumor tissues should be excluded. The PTBE region was segmented on CE-T1WI, T2WI, and ADC maps. For patients with visible preoperative edema, the PTBE region was segmented on the edema region. For those without preoperative edema, the PTBE region was segmented using a width of 0.5 cm along the tumor outline [14].

#### Feature extraction

Based on the image segmentations, quantitative radiomics features were extracted from these ROIs using PyRadiomics (https://github.com/Radiomics/pyradiomics). For every region (TE or PTBE region), 973 radiomics features, including first order statistics, shape-based, gray level co-occurrence matrix (GLCM), gray level size zone matrix (GLSZM), gray level run length matrix (GLRLM), gray level dependence matrix (GLDM), and wavelet-based features were extracted from each sequence.

### Radiomics feature selection and radiomics model building

The Mann–Whitney U test was used to determine the significantly different radiomics features (*p* < 0.05) based on different sequences and combinations of sequences. Then, the Least Absolute Shrinkage and Selection Operator (LASSO) regression was used to select the most informative features (the features with non-zero coefficients). LASSO is a commonly used method for high-dimensional data analysis to improve prediction accuracy and interpretation ability. Finally, A radiomics signature (Rad-Score) was generated by weighting the selected features according to their respective LASSO coefficients in the training set and validation set. Through the support vector machine (SVM) method of training set, radiomics models were constructed from the meaningful features selected from the separate TE region radiomics feature, separate PTBE region radiomics features, and mixed TE region and PTBE region radiomics features, respectively.

### Clinical characteristics analysis

Univariate and multivariate logistic regression (LR) analyses were applied to identify the clinical risk factor of PPCEH after meningioma resection. Results of the multivariate logistics regression analysis of characteristics showing a P-value of less than 0.05 were considered as an independent influence characteristic and were included in the subsequent prediction model construction.

### Construction and validation of clinical and clinical radiomics combined model

LR was applied to construct a clinical model based on all independent influence clinical characteristics, and a clinical radiomics model based on combining the above clinical characteristics with the radiomics features. The clinical radiomics combined model was presented as a nomogram. The prediction performance of the model was evaluated using parameters such as accuracy, specificity, sensitivity, and area under curve (AUC), where the larger AUC value indicated better prediction performance.

### Calibration curve analysis and decision

To estimate the similarity between the nomogram estimated the risk relative to the actual risk of PPCEH, we plotted calibration curves separately for the training and validation sets using the best-performing model. Additionally, decision curve analysis was performed on both the training and validation sets to evaluate the clinical application of the clinical radiomics combined model by assessing the net benefit of the model at different thresholds.

### Statistical analysis

All statistical analyses were performed using R (Version 4.2.2) software. Standardization (Z-score normalization) was performed to balance the deviance of all clinical characteristics and radiomics features. Quantitative variables consistent with a normal distribution were presented as mean ± standard deviation, otherwise, the median (IQR) is used while describing categorical variables as counts and percentages. The differences between subgroups of categorical variables were made using Chi-Square or Fisher Exact tests, while the t-test or Wilcoxon’s test was used for comparisons between quantitative variables.

## Results

### Clinical characteristics

Overall, 148 patients were included, of whom 73 (49.32%) with PPCEH. The clinical characteristics of the different groups are shown in Table 1. The mean age was 54.14 years, and 106 (71.62%) patients were Female. There was no substantial variation in age or gender between the patients with PPCEH and those without PPCEH groups (*p* = 0.78; 0.23). Among all patients, 94 (63.51%) patients had preoperative peritumoral edema, and of those with PPCEH, 60 (82.19%) patients had preoperative peritumoral edema. Compared to patients without preoperative peritumoral edema, those with preoperative peritumoral edema are more likely to develop PPCEH. Additionally, patients with severe tumor adhesion and an unclear tumor-brain interface are also more prone to experiencing PPCEH. Furthermore, patients with PPCEH show higher HbA1c values and intraoperative blood loss compared to those without PPCEH.

**Table 1:** Characteristics of enrolled patients

Characteristics	Whole cohort 148 (100%)	Without PPCEH 75 (50.68%)	PPCEH 73 (49.32%)	*P*-Value
**Gender (%)**				
*Female*	106 (71.62)	55 (73.33)	51 (69.86)	0.78
*Male*	42 (28.38)	20 (26.67)	22 (30.14)	
**Age (mean (SD))**	54.14 (10.93)	53.08 (11.23)	55.23 (10.57)	0.23
**Hypertension (%)**				
*Negative*	117 (79.05)	65 (86.67)	52 (71.23)	0.04
*Positive*	31 (20.95)	10 (13.33)	21 (28.77)	
**Blood Pressure Classification (%)**				
*Normal*	95 (64.19)	50 (66.67)	45 (61.64)	0.55
*Stage 1*	31 (20.95)	15 (20.00)	16 (21.92)	
*Stage 2*	17 (11.49)	9 (12.00)	8 (10.96)	
*Stage 3*	5 (3.38)	1 (1.33)	4 (5.48)	
**HbA1c (median [IQR])**	5.0 [5.0, 5.6]	5.0 [5.0 5.4]	5.1 [5.0, 5.9]	0.01
**Glucose (median [IQR])**	5.16 [4.63, 6.00]	5.10 [4.56, 5.90]	5.30 [4.74, 6.06]	0.14
**Hyperlipidemia (%)**				
*Positive*	41 (27.70)	17 (22.67)	24 (32.88)	0.23
*Negative*	107 (72.30)	58 (77.33)	49 (67.12)	
**Epilepsy (%)**				
*Negative*	130 (87.84)	67 (89.33)	63 (86.30)	0.75
*Positive*	18 (12.16)	8 (10.67)	10 (13.70)	
**WHOCNS (%)**				
*grade I/II (exclude atypical)*	134 (90.54)	67 (89.33)	67 (91.78)	0.82
*atypical grade II/III*	14 (9.46)	8 (10.67)	6 (8.22)	
**Type (%)**				
*Fibrous type*	53 (35.81)	30 (40.00)	23 (31.51)	0.53
*Other*	46 (31.08)	21 (28.00)	25 (34.25)	
*Transitional type*	49 (33.11)	24 (32.00)	25 (34.25)	
**ki67 (median [IQR])**	2.0 [1.0, 5.0]	2.0 [1.0, 3.0]	2.0 [1.0, 5.0]	0.33
**Location (%)**				
*Supratentorial*	113 (76.35)	52 (69.33)	61 (83.56)	0.06
*Sellar region*	18 (12.16)	10 (13.33)	8 (10.96)	
*Infratentorial*	17 (11.49)	13 (17.33)	4 (5.48)	
**Intraoperative Blood Loss** **(median [IQR])**	300 [200, 600]	300 [100, 500]	400 [200, 700]	0.002
**Tumor Adhesion (%)**				
*Severe adhesion*	47 (31.76)	14 (18.67)	33 (45.21)	< 0.001
*No adhesion / mild adhesion*	84 (56.76)	54 (72.00)	30 (41.10)	
*Invasion of bone / venous sinuses*	17 (11.49)	7 (9.33)	10 (13.70)	
**Shape (%)**				
*Lobulated*	27 (18.24)	9 (12.00)	18 (24.66)	0.07
*Round or globular*	73 (49.32)	43 (57.33)	30 (41.10)	
*Irregular*	48 (32.43)	23 (30.67)	25 (34.25)	
**Preoperative peritumoral edema (%)**				
*Positive*	94 (63.51)	34 (45.33)	60 (82.19)	< 0.001
*Negative*	54 (36.49)	41 (54.67)	13 (17.81)	
**Tumor-brain interface (%)**				
*Partially defined borders*	52 (35.14)	16 (21.33)	36 (49.32)	< 0.001
*Poorly defined borders*	16 (10.81)	7 (9.33)	9 (12.33)	
*Well-defined borders*	64 (43.24)	46 (61.33)	18 (24.66)	
*Infiltration into surrounding tissues*	16 (10.81)	6 (8.00)	10 (13.70)	

SD, standard deviation; PPCEH, postoperative progressive cerebral edema and hemorrhage; Categorical variables were presented as the number (percentage). Continuous variables consistent with a normal distribution were presented as mean ± standard deviation, otherwise the median and quartile are used. Chi-Square or Fisher Exact tests, as appropriate, were used to compare the differences in categorical variables, while the independent sample t-test was used to compare the differences in continuous variables, Continuous variables not conforming to the normal distribution were compared by the wilcoxon test

### Logistic regression analysis of clinical characteristics

As shown in Table 2, univariate and multivariate analyses were performed to determine the independent clinical risk features for PPCEH in the training set. We conducted three separate logistic regression analyses based on different training sets obtained from a three-fold cross-validation. In this report, we present the results of one of these analyses. The results of the other two logistic regression analyses are provided in the supplementary Table 2.

**Table 2:** Univariate and multivariate logistics regression analysis of the training set

Character	Univariate analysis		Multivariate analysis	
	OR(95%CI)	*P*-value	OR(95%CI)	*P*-value
**Sex**				
*Female*	-	-		
*Male*	0.92 (0.37–2.3)	0.863		
**Age**	1.01 (0.98–1.05)	0.525		
**Hypertension**				
*Negative*	-	-		
*Positive*	2.1 (0.8–5.8)	0.137		
**Blood Pressure Classification (%)**				
*Normal*				
*Stage 1*	0.82 (0.29–2.23)	0.695		
*Stage 2*	0.6 (0.12–2.65)	0.507		
*Stage 3*	4 (0.56–80.5)	0.226		
**HbA1c**	1.42 (0.95–2.51)	0.15		
**Glucose**	1.3 (0.99–1.83)	0.088		
**Hyperlipidemia**				
*Negative*	-	-		
*Positive*	1.68 (0.71–4.11)	0.244		
**Epilepsy**				
*Negative*	-	-		
*Positive*	1.22 (0.38–4.08)	0.737		
**WHOCNS**				
*grade I/II (exclude atypical)*	-	-		
*atypical grade II/III*	0.65 (0.16–2.44)	0.529		
**Type**				
*Fibrous type*	-	-		
*Other*	1.85 (0.7–5.01)	0.217		
*Transitional type*	1.55 (0.58–4.2)	0.38		
**ki67**	0.97 (0.89–1.05)	0.498		
**Location**				
*Supratentorial*	-	-		
*Infratentorial*	0.69 (0.18–2.49)	0.569	0.63 (0.14–2.79)	0.544
*Sellar region*	0.25 (0.05–0.89)	0.046	0.29 (0.05–1.25)	0.113
**Intraoperative Blood Loss**	1 (1–1)	0.304		
**Tumor Adhesions**				
*No adhesion / mild adhesion*	-	-		
*Severe adhesion*	2.83 (1.18–7.09)	0.022	2.73 (1.00-7.89)	0.054
*Invasion of bone / venous sinuses*	2.83 (0.76–11.93)	0.129	1.35 (0.3–6.5)	0.696
**Shape**				
*Lobulated*	-	-		
*Round or globular*	1.08 (0.3–4.22)	0.908		
*Irregular*	2.12 (0.61–8.05)	0.247		
**Preoperative peritumoral edema (%)**				
*Positive*				
*Negative*	0.17 (0.06–0.41)	< 0.001	0.2 (0.07–0.58)	0.004
**Tumor-brain interface (%)**				
*Partially defined borders*				
*Poorly defined borders*	3.67 (1.44–9.78)	0.007	1.66 (0.55-5)	0.365
*Well-defined borders*	1.33 (0.3–5.47)	0.691	0.62 (0.11–3.13)	0.571
*Infiltration into surrounding tissues*	4.5 (1.24–19.09)	0.028	3.34 (0.71–18.52)	0.140

OR = Odds Ratio, CI = Confidence Interval

We found a significant association between PPCEH and preoperative peritumoral edema (*P* = 0.004), as well as severe tumor adhesion (*P* = 0.054). Compared to the patients with preoperative peritumoral edema, there is a significantly decreased risk of PPCEH in patients without preoperative peritumoral edema (OR 0.20, 95%CI: 0.07–0.58). Compared to the patients with no tumor adhesion or mild adhesion, the patients with severe tumor adhesion were more likely to experience PPCEH after resection of meningioma (OR: 2.73, 95% CI: 1.00-7.89).

### Radiomics feature selection and radiomics features model construction

Through the screening by Mann Whitney U test and LASSO regression, the features that provided the best performance were identified. Rad-score of each patient was calculated based on the above radiomics features and their corresponding LASSO regression coefficients. All rad-scores from different sequences and combinations of sequences in the training set were then separately entered into SVM to build radiomics prediction models. The AUC, ACC, sensitivity, and specificity of various radiomics models to predict PPCEH after meningioma resection are shown in Table 3, where the mean results from the three-fold cross-validation are presented, while the detailed results from each fold are stored in Supplementary Table 3.

**Table 3:** Performance of radiomics features model

Model	Training Set				Validation Set			
	AUC (95%CI)	ACC	Sn	Sp	AUC (95%CI)	ACC	Sn	Sp
**PTBE**								
ADC	0.79 (0.70–0.88)	0.70	0.70	0.71	0.70 (0.54–0.85)	0.64	0.62	0.67
**T2WI**	**0.77** (0.68–0.86)	**0.70**	**0.71**	**0.69**	**0.71** (0.56–0.85)	**0.63**	**0.58**	**0.68**
T1CE	0.76 (0.67–0.86)	0.66	0.65	0.67	0.70 (0.55–0.85)	0.67	0.67	0.67
T2WI + ADC	0.79 (0.70–0.88)	0.72	0.73	0.71	0.70 (0.55–0.85)	0.65	0.63	0.68
ADC + T1CE	0.78 (0.69–0.87)	0.71	0.78	0.65	0.68 (0.53–0.84)	0.63	0.69	0.59
T2WI + T1CE	0.78 (0.69–0.87)	0.70	0.63	0.76	0.68 (0.53–0.83)	0.64	0.58	0.71
ADC + T2WI + T1CE	0.79 (0.71–0.88)	0.71	0.63	0.78	0.69 (0.54–0.84)	0.65	0.59	0.72
**TE**								
ADC	0.74 (0.64–0.84)	0.65	0.53	0.77	0.66 (0.50–0.81)	0.61	0.55	0.67
T2WI	0.81 (0.72–0.89)	0.72	0.66	0.77	0.62 (0.45–0.78)	0.57	0.57	0.59
T1WI	0.87 (0.81–0.94)	0.78	0.75	0.80	0.73 (0.59–0.88)	0.67	0.66	0.68
T1CE	0.75 (0.66–0.85)	0.66	0.69	0.62	0.65 (0.49–0.80)	0.60	0.62	0.59
ADC + T2WI	0.77 (0.68–0.87)	0.69	0.69	0.69	0.55 (0.39–0.72)	0.61	0.63	0.59
ADC + T1CE	0.75 (0.66–0.85)	0.68	0.61	0.75	0.63 (0.47–0.79)	0.59	0.58	0.60
**ADC + T1WI**	**0.83 (0.76–0.91)**	**0.72**	**0.71**	**0.73**	**0.73 (0.58–0.87)**	**0.69**	**0.70**	**0.68**
T2WI + T1WI	0.88 (0.82–0.95)	0.79	0.86	0.72	0.71 (0.56–0.86)	0.65	0.70	0.61
T2WI + T1CE	0.78 (0.69–0.87)	0.69	0.59	0.79	0.59 (0.42–0.75)	0.59	0.55	0.63
T1CE + T1WI	0.85 (0.78–0.91)	0.76	0.74	0.79	0.71 (0.56–0.85)	0.62	0.56	0.68
ADC + T2WI + T1WI	0.88 (0.82–0.95)	0.79	0.84	0.73	0.72 (0.57–0.86)	0.65	0.70	0.60
ADC + T2WI + T1WI	0.78 (0.69–0.87)	0.69	0.75	0.63	0.61 (0.44–0.77)	0.61	0.69	0.55
ADC + T1CE + T1WI	0.80 (0.72–0.89)	0.73	0.70	0.76	0.71 (0.56–0.86)	0.68	0.65	0.72
T2WI + T1CE + T1WI	0.87 (0.80–0.94)	0.79	0.83	0.75	0.73 (0.58–0.87)	0.65	0.69	0.61
ADC + T1CE + T1WI + T2WI	0.88 (0.81–0.94)	0.79	0.86	0.72	0.72 (0.58–0.87)	0.64	0.67	0.61
**PTBE + TE**								
**PTBE: T2WI** **TE: T1WI**	**0.85 (0.78–0.93)**	**0.76**	**0.82**	**0.69**	**0.77 (0.63–0.90)**	**0.67**	**0.67**	**0.67**
PTBE: T2WI TE: ADC + T1WI	0.87 (0.81–0.94)	0.77	0.82	0.71	0.76 (0.62–0.89)	0.67	0.73	0.61

AUC, area under curve; ACC, accuracy; SN, sensitivity; SP, specificity; T2WI, T2-weighted imaging; CE-T1WI, contrast-enhanced T1-weighted imaging; PTBE: peritumoral brain edema; TE: tumor enhancement

The combined model incorporating TE region features from both ADC maps and T1WI shows the best predictive performance among models based on TE region features, the training set mean AUC is 0.83 (95%CI: 0.76–0.91) and the validation set mean AUC is 0.73 (95%CI: 0.58–0.87). When the PTBE region combined with the TE region were considered, the performance of models were further improved, the training set mean AUC is 0.85 (95%CI: 0.78–0.93) and the validation set mean AUC is 0.77 (95%CI: 0.63–0.90). The multi-parameter radiomics model was significantly better than the single-parameter radiomics models.

### Performance of clinical and clinical radiomics combined model

**Fig. 4:**
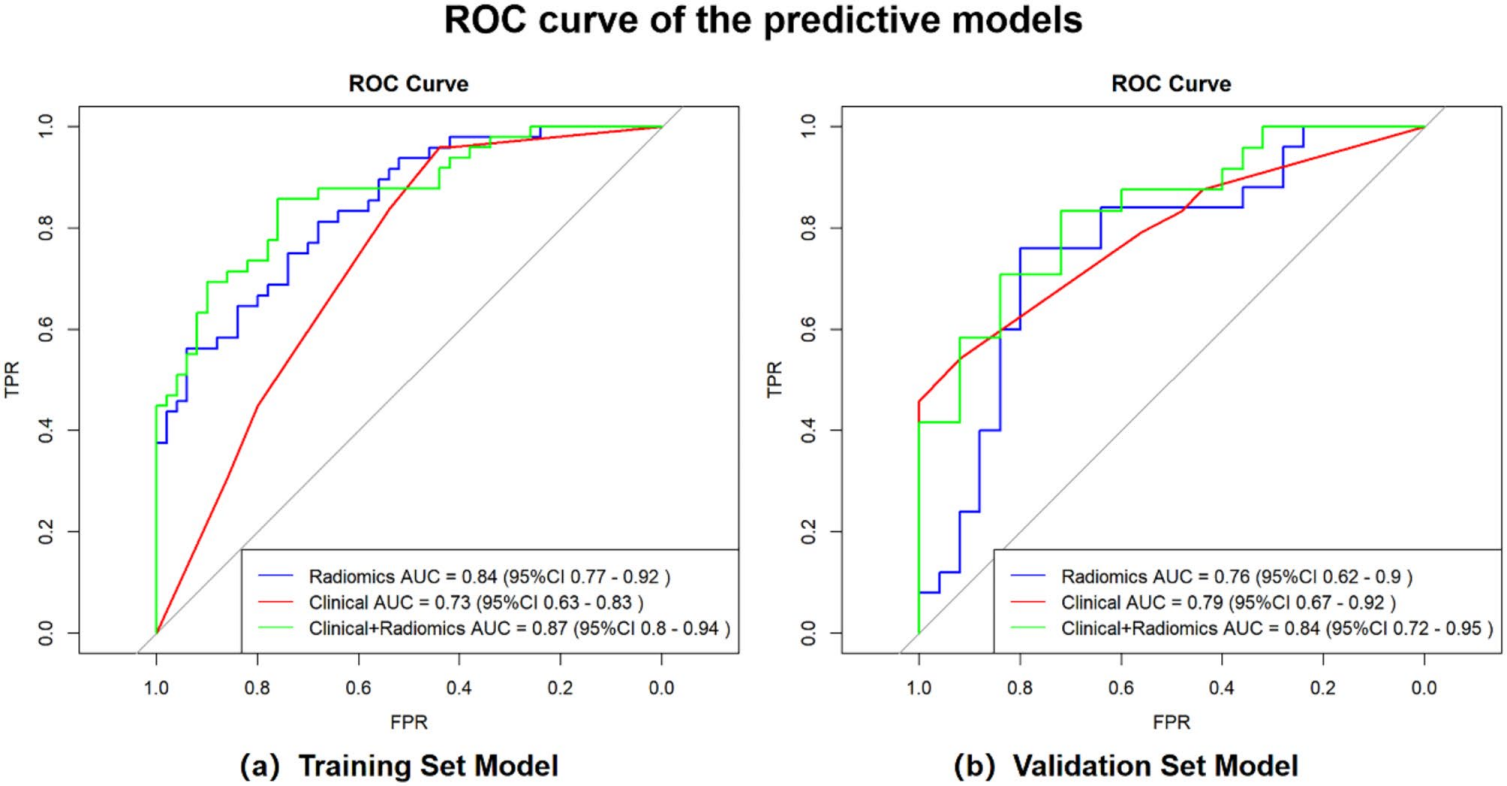
ROC curves of the radiomics model, clinical model, and the combined model in the training and validation sets. The ROC curves for the different models (**a**) in the training set. (**b**) in the validation set

**Fig. 5:**
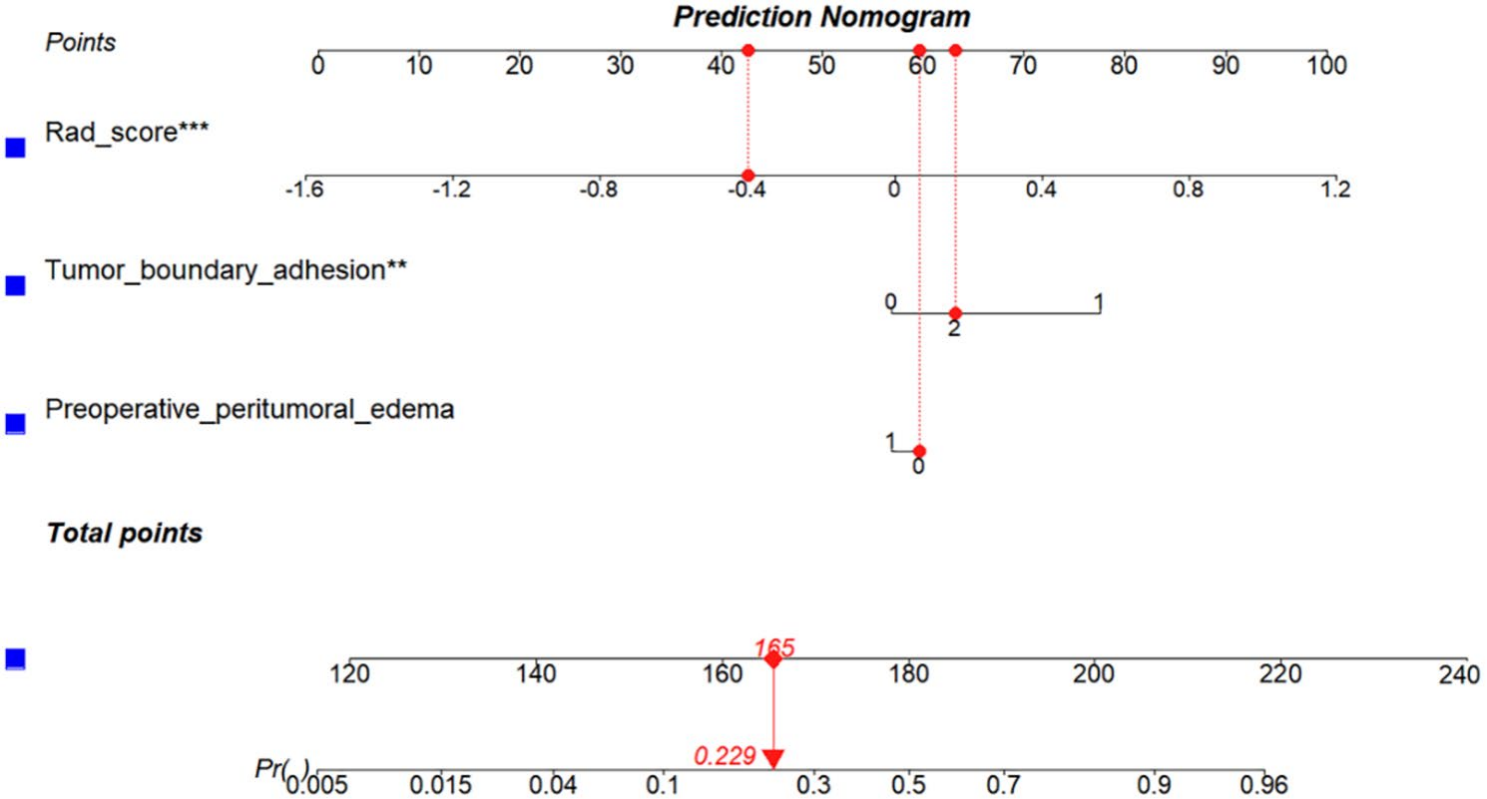
The nomogram for the clinical and radiomics combined model. The Rad-Score, tumor boundary adhesion, and preoperative peritumoral edema were included in the nomogram. PS: Preoperative peritumoral edema: 0 is negative, 1 is positive. Tumor boundary adhesion: 0 is clear boundary/generally adherent, 1 is severe adhesion, 2 is invasion of bone/sinus

The characteristics of preoperative peritumoral edema and tumor adhesion in the training set were used to build the clinical model based on LR and verified the performance of the model in the validation set. As shown in Table 4, the best AUCs from the three-fold cross-validation were 0.73 (95% CI: 0.63–0.83) and 0.79 (95% CI: 0.67–0.92) in the training and validation sets, respectively. Clinical characteristics and Rad-Score from PTBE region combined with the TE region were determined to establish the clinical radiomics combined model, with an AUC of 0.87 (95%CI: 0.80–0.94) in the training set and 0.84 (95%CI: 0.72–0.95) in the validation set from the three-fold cross-validation (Fig. 4). The clinical-radiomics combined model is presented as a nomogram showed in Fig. 5.

**Table 4:** Performance of clinical model, radiomics model, and combined model

Model	Training Set				Validation set			
	AUC (95%CI)	ACC	Sn	Sp	AUC (95%CI)	ACC	Sn	Sp
**Radiomics**								
Fold-1	0.89(0.82–0.95)	0.79	0.92	0.66	0.71(0.56–0.85)	0.59	0.67	0.52
Fold-2	0.83(0.75–0.91)	0.74	0.80	0.68	0.83(0.72–0.94)	0.71	0.79	0.64
Fold-3	0.84(0.77–0.92)	0.74	0.75	0.74	0.76(0.62–0.90)	0.70	0.56	0.84
**Clinical**								
Fold-1	0.73(0.63–0.83)	0.69	0.84	0.54	0.79(0.67–0.92)	0.67	0.79	0.56
Fold-2	0.78(0.69–0.87)	0.69	0.57	0.80	0.69(0.55–0.83)	0.61	0.58	0.64
Fold-3	0.84(0.76–0.92)	0.79	0.77	0.80	0.71(0.56–0.85)	0.62	0.56	0.68
**Clinical + Radiomics**								
Fold-1	0.92(0.86–0.97)	0.86	0.86	0.86	0.69(0.54–0.84)	0.63	0.63	0.64
**Fold-2**	**0.87(0.80–0.94)**	**0.78**	**0.86**	**0.70**	**0.84(0.72–0.95)**	**0.78**	**0.83**	**0.72**
Fold-3	0.87(0.80–0.94)	0.74	0.81	0.68	0.76(0.62–0.90)	0.76	0.76	0.76

AUC, area under curve; ACC, accuracy; SN, sensitivity; SP, specificity

### Calibration and clinical usefulness analysis

**Fig. 6:**
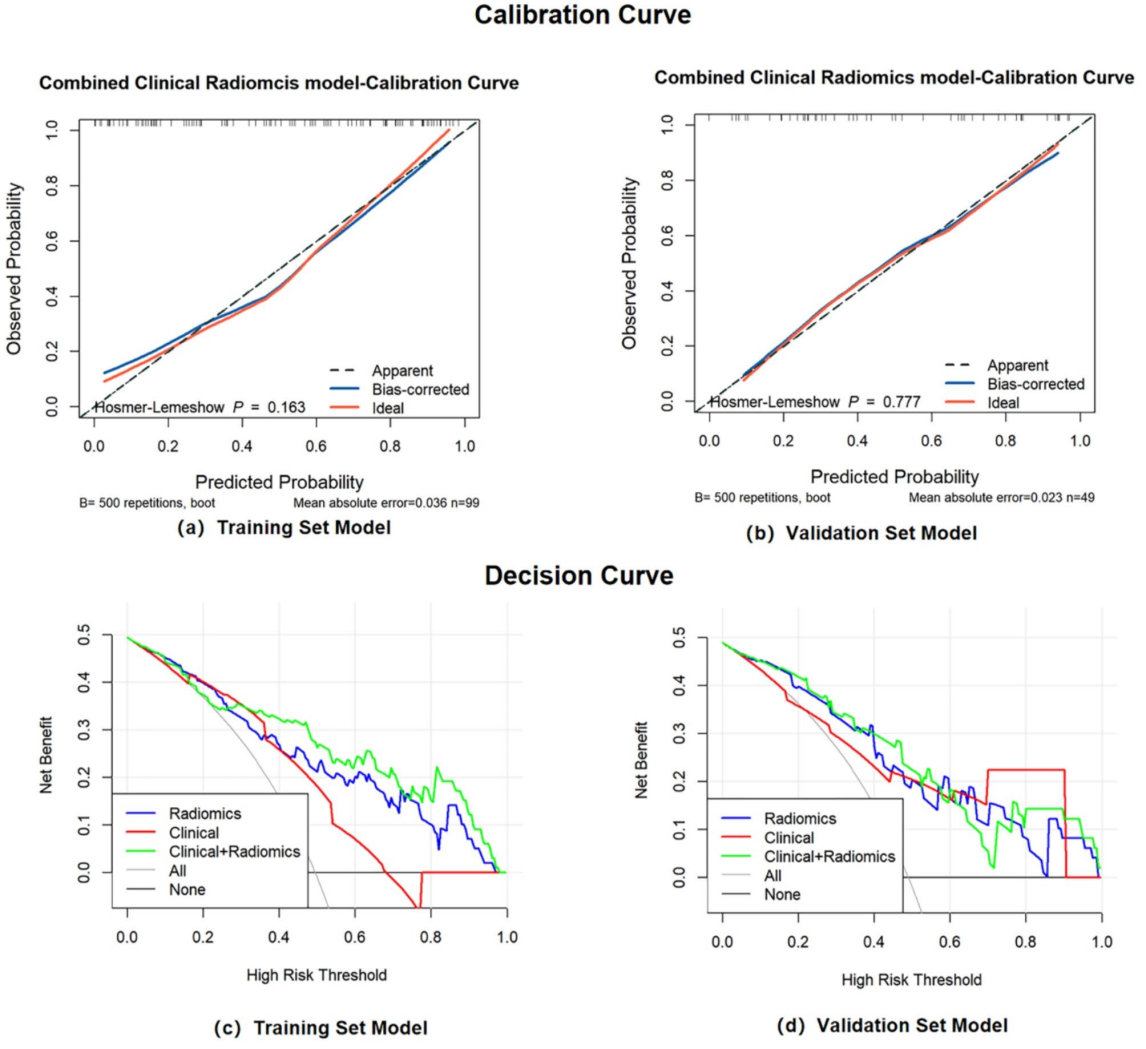
Calibration curve and Decision curve for the clinical and radiomics combined model. The calibration curves of (**a**) the training set and (**b**) the validation set depict the calibration of each model in terms of the agreement between the predicted and actual probability. The decision curves of (**c**) the training set and (**d**) the validation set take the potential consequences of false positives and false negatives into account, aiding the assessment of the net benefit to patients

In both the training sets (*P* = 0.163; Fig. 6a) and validation sets (*P* = 0.777; Fig. 6b), calibration curve analysis and Hosmer-Lemeshow tests for the best clinical radiomics combined model showed good agreement between observations and predictions. The decision curve analysis for the best clinical radiomics combined model is shown in Fig. 6. According to the results, the clinical radiomics combined model had a higher net benefit than either model. The results indicating that the clinical radiomics combined model was clinically useful.

## Discussion

In this study, 73 patients (49.32%) presented PPCEH after resection of meningioma. Our findings demonstrated that the radiomics model using features of both the TE and PTBE regions is better than solely TE regions in predicting PPCEH. Furthermore, the combined model incorporating clinical and radiomics features had the best predictive performance with an AUC of 0.87 in the training set, and 0.84 in the validation set.

The present study shows the incidence of PPCEH as 49.32%. This finding is similar to the previous study by Xiao et al. [9], in which they found 44.1% postoperative cerebral edema exacerbation in 136 patients. Arai et al. reported 33.33% post-operative hemorrhage of meningiomas [15]. In another study of 264 patients with superior parasagittal sinus meningioma [16], 18.4% of patients presented post-operative brain hematoma or cerebral edema. These findings suggest PPCEH are common post-operative complication in patients with meningioma, indicating the necessity and importance of the development of a predictive model in this field. The reasons associated with the higher PPCEH incidence of 49.32% in the presenting study than the published results in the previous studies, may include: (1) the PPCEH consists of two post-operative complications of progressive cerebral edema and progressive cerebral hemorrhage, in contrast, previous studies usually reported the incidence of either post-operative cerebral edema or post-operative cerebral hemorrhage. (2) all the 148 patients enrolled in the presenting study had continuous postoperative follow-up CT and or MRI examinations within one month after surgical resection, which was sensitive to detect the evolution of postoperative cerebral edema and hemorrhage. This imaging acquisition of postoperative continuous follow-up CT and or MRI examinations after resection of meningioma was not applied in the previous studies [7, 9, 16].

In the past decade, multiple clinical studies demonstrated that preoperative PTBE, blood pressure, age, preoperative seizures, and platelet dysfunction, are potential risk factors associated with PPCEH [5, 6, 8, 13]. However, none of these studies established a robust model predicting PPCEH. Recently, radiomics analysis, a novel imaging tool, has been increasingly used in neuro-oncology to predict postoperative complications and post-treatment response after surgical resection of brain tumors [17–19].

Within the published radiomics studies of meningioma, there is no research related to the prediction of post-operative hemorrhage, only one study investigating the prediction of postoperative progressive cerebral edema based on radiomic features of the TE region (training set AUC is 0.86 and validation set AUC is 0.80) [9]. In our study, the radiomic features extracted from the TE region can predict PPCEH (training set AUC is 0.83 and validation set AUC is 0.73), which is similar to the study of Xiao et al. [9].

In the present study, we also found that the radiomics model combing features of TE and PTBE regions improved predictive performance (training set AUC is 0.85 and validation set AUC is 0.77) than the radiomics model based on TE regions only. This finding is similar to the previous studies which reported that a combination of both TE and PTBE regions could grade meningioma better [20] or increase predictive performance of brain invasion in meningioma [21]. Our findings suggest combination radiomics features from PTBE and TE regions can enhance the performance of predictive models of post-operative complications, and indicate the extension of tumor feature sources for radiomics analysis in brain tumors.

Within the radiomics analysis of our study, we found that the features extracted from the PTBE region on the ADC map (training set AUC is 0.70, validation set AUC: 0.70), on CE-T1WI (training set AUC is 0.66, validation set AUC: 0.70), on T2WI (training set AUC is 0.70, validation set AUC: 0.71) showed similar predictive performance. Few of published radiomics study in meningioma analyzed PTBE features of ADC [20]. Wang et al. reported PTBE features on CE-T1WI had better performance in grading gliomas than the PTBE features of ADC [22]. Depending on the measurement of the movement of water protons in cellular spaces (Brownian motion), ADC can provide quantitative functional information of both enhancing meningiomas and their PTBE compared to conventional MR sequences. Deeper mining of such functional features of ADC or application of more advanced MRI sequences in radiomics models can be identified in future artificial intelligence studies in meningiomas.

Our findings also suggest that PTBE may play a vital role in the development of PPCEH after the resection of meningioma. This proposes new insights into the pathogenesis of PPCEH, subsequently leading to a better understanding of the mechanisms of PPCEH. The establishment of a combined pre-operative MR multi-parameter radiomics model incorporating both TE and PTBE radiomics features of our study can non-invasively and accurately predict PPCEH after resection of meningioma. Accurate prediction of PPCEH not only is useful for pre-operative personalized treatment decisions and post-operative clinical management but also improves the quality of life in such patients with meningioma.

This study has several limitations. Firstly, the sample size of the present study was relatively small. Secondly, our study enrolled patients from a single center and no external validation was performed. Therefore, further study including a large population from multiple centers will be performed in the future.

## Conclusions

We developed an innovative machine learning based MRI radiomics model which can accurately predict PPCEH after resection of meningioma. Our findings demonstrated PTBE region may play a pivotal role in PPCEH, which can propose new insights into a better understanding of the mechanisms of PPCEH and will be useful for the improvement of personalized treatment and quality of life in patients with meningioma.

## Electronic supplementary material

Below is the link to the electronic supplementary material.

Supplementary Material 1

Supplementary Material 2

Supplementary Material 3

## Data Availability

The data and code used in this study are publicly available on GitHub at the following link: https://github.com/tanamian/MeningiomaMRI-Radiomics. Readers can access the relevant datasets and analysis code at this URL.

## Abbreviations

CNS: Central nervous system
PPCEH: Postoperative progressive cerebral edema and hemorrhage
TE: Tumor enhancement
PTBE: Peritumoral brain edema
SBP: Systolic blood pressure
DBP: Diastolic blood pressure
TIWI: T1-weighted imaging
CE-T1WI: Contrast-enhanced T1-weighted imaging
DWI: Diffusion-weighted imaging
ADC: Apparent diffusion coefficient
T2WI: T2-weighted imaging
GLCM: Gray level co-occurrence matrix
GLSZM: Gray level size zone matrix
GLRLM: Gray level run length matrix
GLDM: Gray level dependence matrix
LASSO: Least absolute shrinkage and selection operator
SVM: Support vector machine
LR: Logistic regression
AUC: Area under curve
