# Supplementary material for: Multi-parameter MRI radiomics model in predicting postoperative progressive cerebral edema and hemorrhage after resection of meningioma
Source: Cancer Imaging. 2024 Nov 1;24:149. doi: 10.1186/s40644-024-00796-3 (PMC11529156; doi:10.1186/s40644-024-00796-3)
Supplement: Supplementary file 1 — Supplementary Material 1 [file 40644_2024_796_MOESM1_ESM.docx]

**Supplementary Table S1：MRI Scan Parameter**

| **Sequences** | **Parameter** |
| --- | --- |
| **T1WI** | TR/TE=2 036.48ms/20.16ms ， TI=720ms ， NEX 1.0 ， slice thickness 5mm ， FOV=220mm×220mm，matrix 320×224； |
| **T2WI** | TR/TE=4 400 ms/102.72ms，TI=0ms，NEX 2.0，slice thickness 5mm，FOV =220mm×220mm，matrix 288×192； |
| **DWI** | TR/TE=5 025 ms/78.8ms，b=0,1000 s/mm²，NEX 2.0，slice thickness 5mm，FOV =240mm×240mm，matrix 128×96 |
| **T1CE** | TR/TE=9.25ms/3.65ms，TI=350ms，NEX 1.0，slice thickness 1.8mm，FOV =250mm×250mm，matrix 256×224； |
