# Supplementary material for: Multi-parameter MRI radiomics model in predicting postoperative progressive cerebral edema and hemorrhage after resection of meningioma
Source: Cancer Imaging. 2024 Nov 1;24:149. doi: 10.1186/s40644-024-00796-3 (PMC11529156; doi:10.1186/s40644-024-00796-3)
Supplement: Supplementary file 2 — Supplementary Material 2 [file 40644_2024_796_MOESM2_ESM.docx]

**Supplementary Table 2-1：**

**Univariate and multivariate logistics regression analysis of the training set**

| **Character** | **Univariate analysis** | | **Multivariate analysis** | |
| --- | --- | --- | --- | --- |
|  | **OR(95%CI)** | **P-value** | **OR(95%CI)** | **P-value** |
| **Sex** |  |  |  |  |
| *Female* |  |  |  |  |
| *Male* | 1.42 (0.57-3.59) | 0.453 |  |  |
| **Age** | 1.03 (0.99-1.07) | 0.179 |  |  |
| **Hypertension** |  |  |  |  |
| *Negative* |  |  |  |  |
| *Positive* | 2.93 (1.06-9.01) | 0.045 | 3.17 (0.88-12.61) | 0.086 |
| **Blood Pressure Classification (%)** |  |  |  |  |
| *Normal* |  |  |  |  |
| *Stage 1* | 1.77 (0.64-5.1) | 0.277 |  |  |
| *Stage 2* | 0.88 (0.26-2.85) | 0.837 |  |  |
| *Stage 3* | 3.54 (0.43-73.69) | 0.286 |  |  |
| **HbA1c** | 1.47 (1.01-2.54) | 0.096 |  |  |
| **Glucose** | 1.03 (0.81-1.33) | 0.8 |  |  |
| **Hyperlipidemia** |  |  |  |  |
| *Negative* |  |  |  |  |
| *Positive* | 2.25 (0.94-5.56) | 0.072 |  |  |
| **Epilepsy** |  |  |  |  |
| *Negative* |  |  |  |  |
| *Positive* | 1.6 (0.43-6.64) | 0.486 |  |  |
| **WHOCNS** |  |  |  |  |
| *grade I/II (exclude atypical)* |  |  |  |  |
| *atypical grade II/III* | 0.55 (0.14-1.94) | 0.361 |  |  |
| **Type** |  |  |  |  |
| *Fibrous type* |  |  |  |  |
| *Other* | 1.25 (0.5-3.19) | 0.633 |  |  |
| *Transitional type* | 0.84 (0.31-2.29) | 0.739 |  |  |
| **ki67** | 0.99 (0.92-1.06) | 0.738 |  |  |
| **Location** |  |  |  |  |
| *Supratentorial* |  |  |  |  |
| *Infratentorial* | 0.66 (0.21-1.96) | 0.452 |  |  |
| *Sellar region* | 0.32 (0.07-1.2) | 0.109 |  |  |
| **Intraoperative Blood Loss** | 1 (1-1) | 0.02 | 1 (1-1) | 0.270 |
| **Tumor Adhesions** |  |  |  |  |
| *No adhesion / mild adhesion* |  |  |  |  |
| *Severe adhesion* | 5.97 (2.27-17.38) | <0.001 | 6.66 (2.05-24.66) | 0.003 |
| *Invasion of bone / venous sinuses* | 2.66 (0.75-10.03) | 0.131 | 1.65 (0.33-8.29) | 0.538 |
| **Shape** |  |  |  |  |
| *Lobulated* |  |  |  |  |
| *Round or globular* | 4.27 (1.35-15.26) | 0.017 |  |  |
| *Irregular* | 2.6 (1.06-6.6) | 0.04 |  |  |
| **Preoperative peritumoral edema (%)** |  |  |  |  |
| *Positive* |  |  |  |  |
| *Negative* | 0.18 (0.07-0.42) | <0.001 | 0.31 (0.08-1.13) | 0.078 |
| **Tumor-brain interface (%)** |  |  |  |  |
| *Partially defined borders* |  |  |  |  |
| *Poorly defined borders* | 5.64 (2.18-15.54) | <0.001 | 1.53 (0.39-5.74) | 0.532 |
| *Well-defined borders* | 5.64 (1.48-24.82) | 0.014 | 2.55 (0.41-17.08) | 0.317 |
| *Infiltration into surrounding tissues* | 5.64 (1.27-30.56) | 0.028 | 4.71 (0.74-33.72) | 0.104 |

**Supplementary Table 2-2：**

**Univariate and multivariate logistics regression analysis of the training set**

| **Character** | **Univariate analysis** | | **Multivariate analysis** | |
| --- | --- | --- | --- | --- |
|  | **OR(95%CI)** | **P-value** | **OR(95%CI)** | **P-value** |
| **Sex** |  |  |  |  |
| *Female* |  |  |  |  |
| *Male* | 1.27 (0.55-2.96) | 0.568 |  |  |
| **Age** | 1.02 (0.98-1.06) | 0.332 |  |  |
| **Hypertension** |  |  |  |  |
| *Negative* |  |  |  |  |
| *Positive* | 3.02 (1.09-9.29) | 0.04 | 2.41 (0.61-10.23) | 0.215 |
| **Blood Pressure Classification (%)** |  |  |  |  |
| *Normal* |  |  |  |  |
| *Stage 1* | 1.17 (0.44-3.13) | 0.748 |  |  |
| *Stage 2* | 1.64 (0.47-6.07) | 0.437 |  |  |
| *Stage 3* | NA | 0.991 |  |  |
| **HbA1c** | 4.14 (1.69-12.5) | 0.006 | 3.59 (1.42-13.4) | 0.033 |
| **Glucose** | 1.28 (1-1.75) | 0.08 |  |  |
| **Hyperlipidemia** |  |  |  |  |
| *Negative* |  |  |  |  |
| *Positive* | 1.18 (0.46-3.05) | 0.726 |  |  |
| **Epilepsy** |  |  |  |  |
| *Negative* |  |  |  |  |
| *Positive* | 1.25 (0.39-4.19) | 0.707 |  |  |
| **WHOCNS** |  |  |  |  |
| *grade I/II (exclude atypical)* |  |  |  |  |
| *atypical grade II/III* | 1.42 (0.3-7.57) | 0.655 |  |  |
| **Type** |  |  |  |  |
| *Fibrous type* |  |  |  |  |
| *Other* | 1.6 (0.56-4.64) | 0.38 |  |  |
| *Transitional type* | 1.81 (0.73-4.6) | 0.204 |  |  |
| **ki67** | 0.99 (0.92-1.07) | 0.886 |  |  |
| **Location** |  |  |  |  |
| *Supratentorial* |  |  |  |  |
| *Infratentorial* | 0.7 (0.16-2.85) | 0.621 |  |  |
| *Sellar region* | 0.22 (0.03-0.95) | 0.066 |  |  |
| **Intraoperative Blood Loss** | 1 (1-1) | 0.295 |  |  |
| **Tumor Adhesions** |  |  |  |  |
| *No adhesion / mild adhesion* |  |  |  |  |
| *Severe adhesion* | 4.84 (1.93-13.05) | 0.001 | 3.19 (1.02-10.47) | 0.048 |
| *Invasion of bone / venous sinuses* | 2.27 (0.61-8.84) | 0.219 | 0.47 (0.08-2.66) | 0.395 |
| **Shape** |  |  |  |  |
| *Lobulated* |  |  |  |  |
| *Round or globular* | 2.93 (1.03-9.01) | 0.05 |  |  |
| *Irregular* | 1.13 (0.45-2.84) | 0.799 |  |  |
| **Preoperative peritumoral edema (%)** |  |  |  |  |
| *Positive* |  |  |  |  |
| *Negative* | 0.18 (0.07-0.47) | <0.001 | 0.25 (0.06-0.95) | 0.047 |
| **Tumor-brain interface (%)** |  |  |  |  |
| *Partially defined borders* |  |  |  |  |
| *Poorly defined borders* | 9.75 (3.57-29.34) | <0.001 | 3.7 (0.99-14.51) | 0.054 |
| *Well-defined borders* | 4.5 (1.09-20.58) | 0.04 | 1.01 (0.15-6.32) | 0.994 |
| *Infiltration into surrounding tissues* | 3 (0.71-12.81) | 0.128 | 1.19 (0.2-6.61) | 0.841 |
