# Supplementary material for: Multi-parameter MRI radiomics model in predicting postoperative progressive cerebral edema and hemorrhage after resection of meningioma
Source: Cancer Imaging. 2024 Nov 1;24:149. doi: 10.1186/s40644-024-00796-3 (PMC11529156; doi:10.1186/s40644-024-00796-3)
Supplement: Supplementary file 3 — Supplementary Material 3 [file 40644_2024_796_MOESM3_ESM.docx]

**Supplementary Table 3: Performance Table of Different Radiomics Models under Three-Fold Cross-Validation**

| **Model** | **Folds** | **Training Set** | | | | **Validation Set** | | | |
| --- | --- | --- | --- | --- | --- | --- | --- | --- | --- |
|  |  | **AUC (95%CI)** | **ACC** | **Sn** | **Sp** | **AUC (95%CI)** | **ACC** | **Sn** | **Sp** |
| **PTBE** |  |  |  |  |  |  |  |  |  |
| ADC | 1 | 0.78 (0.69-0.87) | 0.67 | 0.80 | 0.54 | 0.67 (0.51-0.83) | 0.65 | 0.75 | 0.56 |
|  | 2 | 0.83 (0.75-0.91) | 0.76 | 0.67 | 0.84 | 0.69 (0.53-0.84) | 0.63 | 0.54 | 0.72 |
|  | 3 | 0.75 (0.65-0.85) | 0.68 | 0.63 | 0.74 | 0.73 (0.59-0.87) | 0.64 | 0.56 | 0.72 |
| T2WI | 1 | 0.80 (0.71-0.89) | 0.71 | 0.82 | 0.60 | 0.68 (0.52-0.83) | 0.61 | 0.67 | 0.56 |
|  | 2 | 0.73 (0.63-0.83) | 0.70 | 0.67 | 0.72 | 0.73 (0.59-0.87) | 0.63 | 0.63 | 0.64 |
|  | 3 | 0.77 (0.68-0.86) | 0.68 | 0.63 | 0.74 | 0.71 (0.57-0.86) | 0.64 | 0.44 | 0.84 |
| T1CE | 1 | 0.73 (0.63-0.83) | 0.64 | 0.76 | 0.52 | 0.75 (0.61-0.88) | 0.69 | 0.83 | 0.56 |
|  | 2 | 0.79 (0.71-0.88) | 0.69 | 0.65 | 0.72 | 0.61 (0.45-0.77) | 0.63 | 0.67 | 0.60 |
|  | 3 | 0.76 (0.66-0.85) | 0.66 | 0.54 | 0.78 | 0.74 (0.60-0.88) | 0.68 | 0.52 | 0.84 |
| T2WI+ADC | 1 | 0.82 (0.73-0.90) | 0.72 | 0.82 | 0.62 | 0.68 (0.53-0.84) | 0.65 | 0.71 | 0.60 |
|  | 2 | 0.79 (0.70-0.88) | 0.73 | 0.65 | 0.80 | 0.72 (0.57-0.86) | 0.63 | 0.58 | 0.68 |
|  | 3 | 0.77 (0.68-0.86) | 0.71 | 0.71 | 0.72 | 0.71 (0.57-0.86) | 0.68 | 0.60 | 0.76 |
| ADC+T1CE | 1 | 0.79 (0.70-0.88) | 0.73 | 0.90 | 0.56 | 0.68 (0.52-0.84) | 0.63 | 0.79 | 0.48 |
|  | 2 | 0.78 (0.69-0.87) | 0.73 | 0.80 | 0.66 | 0.62 (0.46-0.78) | 0.59 | 0.75 | 0.44 |
|  | 3 | 0.76 (0.67-0.86) | 0.68 | 0.63 | 0.74 | 0.75 (0.62-0.89) | 0.68 | 0.52 | 0.84 |
| T2WI+T1CE | 1 | 0.79 (0.71-0.88) | 0.72 | 0.73 | 0.70 | 0.68 (0.52-0.83) | 0.63 | 0.67 | 0.60 |
|  | 2 | 0.79 (0.70-0.88) | 0.70 | 0.63 | 0.76 | 0.62 (0.45-0.78) | 0.63 | 0.63 | 0.64 |
|  | 3 | 0.76 (0.67-0.86) | 0.68 | 0.54 | 0.82 | 0.75 (0.61-0.89) | 0.66 | 0.44 | 0.88 |
| ADC+T2WI+T1CE | 1 | 0.81 (0.73-0.90) | 0.73 | 0.71 | 0.74 | 0.69 (0.53-0.84) | 0.67 | 0.67 | 0.68 |
|  | 2 | 0.81 (0.73-0.89) | 0.71 | 0.65 | 0.76 | 0.64 (0.47-0.80) | 0.63 | 0.67 | 0.60 |
|  | 3 | 0.76 (0.67-0.86) | 0.69 | 0.54 | 0.84 | 0.75 (0.61-0.89) | 0.66 | 0.44 | 0.88 |
| **TE** |  |  |  |  |  |  |  |  |  |
| ADC | 1 | 0.74 (0.65-0.84) | 0.61 | 0.55 | 0.66 | 0.62 (0.46-0.78) | 0.57 | 0.63 | 0.52 |
|  | 2 | 0.72 (0.63-0.82) | 0.65 | 0.55 | 0.74 | 0.71 (0.56-0.86) | 0.65 | 0.63 | 0.68 |
|  | 3 | 0.75 (0.66-0.85) | 0.69 | 0.48 | 0.90 | 0.64 (0.48-0.79) | 0.60 | 0.40 | 0.80 |
| T2WI | 1 | 0.76 (0.66-0.85) | 0.70 | 0.67 | 0.72 | 0.57 (0.40-0.74) | 0.59 | 0.71 | 0.48 |
|  | 2 | 0.82 (0.74-0.91) | 0.74 | 0.82 | 0.66 | 0.64 (0.48-0.80) | 0.59 | 0.63 | 0.56 |
|  | 3 | 0.84 (0.76-0.91) | 0.71 | 0.48 | 0.94 | 0.64 (0.48-0.79) | 0.54 | 0.36 | 0.72 |
| T1WI | 1 | 0.95 (0.93-0.99) | 0.88 | 0.86 | 0.90 | 0.69 (0.53-0.84) | 0.63 | 0.71 | 0.56 |
|  | 2 | 0.81 (0.70-0.87) | 0.68 | 0.67 | 0.68 | 0.82 (0.65-0.91) | 0.73 | 0.79 | 0.68 |
|  | 3 | 0.87 (0.81-0.94) | 0.78 | 0.79 | 0.76 | 0.74 (0.60-0.88) | 0.70 | 0.68 | 0.72 |
| T1CE | 1 | 0.74 (0.65-0.84) | 0.66 | 0.80 | 0.52 | 0.60 (0.44-0.76) | 0.59 | 0.83 | 0.36 |
|  | 2 | 0.71 (0.61-0.81) | 0.61 | 0.61 | 0.60 | 0.72 (0.57-0.87) | 0.67 | 0.58 | 0.76 |
|  | 3 | 0.81 (0.72-0.89) | 0.70 | 0.67 | 0.74 | 0.62 (0.46-0.78) | 0.54 | 0.44 | 0.64 |
| ADC+T2WI | 1 | 0.75 (0.65-0.84) | 0.69 | 0.73 | 0.64 | 0.58 (0.41-0.75) | 0.59 | 0.75 | 0.44 |
|  | 2 | 0.82 (0.75-0.90) | 0.69 | 0.84 | 0.54 | 0.66 (0.50-0.81) | 0.65 | 0.75 | 0.56 |
|  | 3 | 0.75 (0.66-0.85) | 0.69 | 0.50 | 0.88 | 0.42(0.26-0.58) | 0.58 | 0.40 | 0.76 |
| ADC+T1CE | 1 | 0.78 (0.68-0.87) | 0.72 | 0.80 | 0.64 | 0.60 (0.44-0.76) | 0.53 | 0.75 | 0.32 |
|  | 2 | 0.72 (0.62-0.82) | 0.63 | 0.57 | 0.68 | 0.71 (0.56-0.86) | 0.67 | 0.71 | 0.64 |
|  | 3 | 0.76 (0.67-0.86) | 0.70 | 0.46 | 0.94 | 0.58 (0.42-0.74) | 0.56 | 0.28 | 0.84 |
| ADC+T1WI | 1 | 0.85 (0.77-0.92) | 0.78 | 0.80 | 0.76 | 0.69 (0.54-0.84) | 0.67 | 0.71 | 0.64 |
|  | 2 | 0.74 (0.65-0.84) | 0.63 | 0.69 | 0.56 | 0.76 (0.62-0.89) | 0.67 | 0.79 | 0.56 |
|  | 3 | 0.91 (0.86-0.97) | 0.77 | 0.65 | 0.88 | 0.73 (0.59-0.88) | 0.72 | 0.60 | 0.84 |
| T2WI+T1WI | 1 | 0.86 (0.79-0.94) | 0.79 | 0.88 | 0.70 | 0.69 (0.53-0.84) | 0.63 | 0.79 | 0.48 |
|  | 2 | 0.90 (0.84-0.96) | 0.80 | 0.94 | 0.66 | 0.75 (0.61-0.89) | 0.71 | 0.83 | 0.60 |
|  | 3 | 0.88 (0.82-0.95) | 0.78 | 0.75 | 0.80 | 0.70 (0.55-0.85) | 0.62 | 0.48 | 0.76 |
| T2WI+T1CE | 1 | 0.75 (0.65-0.84) | 0.66 | 0.63 | 0.68 | 0.58 (0.41-0.75) | 0.59 | 0.71 | 0.48 |
|  | 2 | 0.84 (0.76-0.91) | 0.75 | 0.71 | 0.78 | 0.63 (0.47-0.79) | 0.61 | 0.58 | 0.64 |
|  | 3 | 0.76 (0.66-0.85) | 0.66 | 0.42 | 0.90 | 0.56 (0.39-0.72) | 0.56 | 0.36 | 0.76 |
| T1CE+T1WI | 1 | 0.97 (0.94-1.00) | 0.91 | 0.94 | 0.88 | 0.64 (0.49-0.80) | 0.59 | 0.67 | 0.52 |
|  | 2 | 0.74 (0.65-0.84) | 0.64 | 0.59 | 0.68 | 0.79(0.66-0.92) | 0.63 | 0.54 | 0.72 |
|  | 3 | 0.82 (0.74-0.90) | 0.74 | 0.69 | 0.80 | 0.69 (0.54-0.84) | 0.64 | 0.48 | 0.80 |
| ADC+T2WI+T1WI | 1 | 0.86 (0.79-0.94) | 0.80 | 0.88 | 0.72 | 0.68 (0.53-0.83) | 0.63 | 0.79 | 0.48 |
|  | 2 | 0.89 (0.83-0.95) | 0.80 | 0.90 | 0.70 | 0.76 (0.62-0.90) | 0.67 | 0.83 | 0.52 |
|  | 3 | 0.90 (0.85-0.96) | 0.76 | 0.73 | 0.78 | 0.71 (0.56-0.85) | 0.64 | 0.48 | 0.80 |
| ADC+T2WI+T1CE | 1 | 0.74 (0.65-0.84) | 0.69 | 0.84 | 0.54 | 0.58 (0.41-0.75) | 0.61 | 0.79 | 0.44 |
|  | 2 | 0.81 (0.72-0.89) | 0.69 | 0.88 | 0.50 | 0.67 (0.52-0.83) | 0.67 | 0.88 | 0.48 |
|  | 3 | 0.79 (0.71-0.88) | 0.69 | 0.54 | 0.84 | 0.57 (0.41-0.73) | 0.56 | 0.40 | 0.72 |
| ADC+T1CE+T1WI | 1 | 0.86 (0.78-0.94) | 0.83 | 0.86 | 0.80 | 0.70 (0.55-0.85) | 0.67 | 0.75 | 0.60 |
|  | 2 | 0.71 (0.61-0.81) | 0.61 | 0.53 | 0.68 | 0.76 (0.63-0.90) | 0.71 | 0.71 | 0.72 |
|  | 3 | 0.84 (0.76-0.91) | 0.76 | 0.71 | 0.80 | 0.67 (0.52-0.82) | 0.66 | 0.48 | 0.84 |
| T2WI+T1CE+T1WI | 1 | 0.87 (0.79-0.94) | 0.80 | 0.88 | 0.72 | 0.70 (0.55-0.85) | 0.65 | 0.79 | 0.52 |
|  | 2 | 0.86 (0.79-0.93) | 0.77 | 0.84 | 0.70 | 0.75 (0.61-0.89) | 0.67 | 0.79 | 0.56 |
|  | 3 | 0.88 (0.82-0.94) | 0.80 | 0.77 | 0.82 | 0.73 (0.58-0.87) | 0.62 | 0.48 | 0.76 |
| ADC+T1CE+T1WI+T2WI | 1 | 0.86 (0.78-0.93) | 0.79 | 0.88 | 0.70 | 0.70 (0.55-0.85) | 0.63 | 0.79 | 0.48 |
|  | 2 | 0.86 (0.79-0.93) | 0.79 | 0.90 | 0.68 | 0.73 (0.59-0.88) | 0.63 | 0.75 | 0.52 |
|  | 3 | 0.91 (0.86-0.96) | 0.79 | 0.79 | 0.78 | 0.74 (0.59-0.88) | 0.66 | 0.48 | 0.84 |
| **PTBE+TE** |  |  |  |  |  |  |  |  |  |
| PTBE: T2WI  TE: T1WI | 1 | 0.89 (0.82-0.95) | 0.79 | 0.92 | 0.66 | 0.71 (0.56-0.85) | 0.59 | 0.67 | 0.52 |
|  | 2 | 0.83 (0.75-0.91) | 0.74 | 0.80 | 0.68 | 0.83 (0.72-0.94) | 0.71 | 0.79 | 0.64 |
|  | 3 | 0.84 (0.77-0.92) | 0.74 | 0.75 | 0.74 | 0.76 (0.62-0.90) | 0.70 | 0.56 | 0.84 |
| PTBE: T2WI  TE: T1WI+ADC | 1 | 0.87 (0.80-0.94) | 0.74 | 0.88 | 0.60 | 0.69 (0.54-0.84) | 0.59 | 0.71 | 0.48 |
|  | 2 | 0.84 (0.76-0.91) | 0.77 | 0.80 | 0.74 | 0.84 (0.73-0.95) | 0.73 | 0.88 | 0.60 |
|  | 3 | 0.92 (0.87-0.97) | 0.80 | 0.79 | 0.80 | 0.74 (0.60-0.88) | 0.68 | 0.60 | 0.76 |
